# Effective doses of remimazolam and esketamine combined with remifentanil for endotracheal intubation without muscle relaxants in pediatric patients

**DOI:** 10.3389/fphar.2025.1558966

**Published:** 2025-03-18

**Authors:** Jinming Chen, Ying Mai, Xiaolei Cheng, Hao Sun, Zhihong Chen, Zhongqi Zhang

**Affiliations:** Department of Anesthesiology, The Affiliated Shunde Hospital of Jinan University, Foshan, China

**Keywords:** remimazolam, esketamine, effective dose, without muscle relaxants, dixon’s up-and-down method

## Abstract

**Introduction:**

The combination of remimazolam and esketamine effectively alleviates adverse hemodynamic effects, such as tachycardia and hypertension, during intubation. However, the dosage for achieving optimal intubation conditions when co-administered with remifentanil remains unestablished. Therefore, this study aimed to determine the effective doses of remimazolam and esketamine for endotracheal intubation without muscle relaxants in pediatric patients using Dixon’s up-and-down method.

**Methods:**

This prospective, non-controlled, non-randomized clinical trial sequentially allocated 41 children aged 3–6 into two phases. All patients underwent tracheal intubation under general anesthesia. Patients received a fixed dose of remifentanil at 2.5 μg/kg via a pump over 90 s. In the first phase, the induction dose of remimazolam was set at 0.2 mg/kg. The first patient received esketamine at a dose of 0.5 mg/kg, administered with a dose gradient of 0.2 mg/kg based on Dixon’s up-and-down method. 50% effective dose (ED_50_) and 95% effective dose (ED_95_) for esketamine were then measured through probit regression analysis. Similarly, in the second phase, the ED_95_ of esketamine was fixed. The first patient received remimazolam at a dose of 0.2 mg/kg, administered at a dose gradient of 0.1 mg/kg. ED_50_ and ED_95_ for remimazolam were then measured. Intubation conditions were assessed via the Copenhagen scale. Heart rate (HR) and mean arterial pressure (MAP) were recorded at the following time points: Just before intubation (T1) and 1 min after intubation (T2). Adverse events were also recorded during anesthesia induction.

**Results:**

At a fixed dose of remifentanil (2.5 μg/kg), the ED_50_ of esketamine was 0.74 mg/kg (95% confidence interval [CI]: 0.61–0.89 mg/kg), while the ED_95_ was 0.97 mg/kg (95% CI: 0.85–1.75 mg/kg). The ED_50_ of remimazolam was 0.39 mg/kg (95% CI: 0.29–0.53 mg/kg), while the ED_95_ was 0.56 mg/kg (95% CI: 0.46–1.47 mg/kg). Hemodynamic stability was maintained during anesthesia induction, with no significant adverse events observed.

**Conclusion:**

The ED_50_ and ED_95_ values of remimazolam and esketamine in this study provide initial dosing references for pediatric endotracheal intubation without muscle relaxants. A fixed dose of 2.5 μg/kg remifentanil combined with these agents is safe and effective in children aged 3–6 years, though further multicenter studies are recommended for validation.

**Clinical trial registration:**

www.chictr.org.cn, number: ChiCTR2200063847.

## 1 Introduction

Endotracheal intubation is crucial in pediatric anesthesia, often requiring sedatives and analgesics to facilitate intubation and reduce patient discomfort. Muscle relaxants aid pediatric tracheal intubation but risk prolonged paralysis and neuromuscular recovery issues (Tamire et al., 2021). Intubation without muscle relaxants has recently been explored as an alternative approach, with many studies demonstrating that omitting muscle relaxants during pediatric anesthesia intubation can effectively alleviate muscular discomfort and expedite postoperative recovery (Karanth et al., 2018; Naziri et al., 2015; Rizvanović et al., 2017).

Remimazolam, a fast-acting benzodiazepine, offers quick onset and recovery, and can be swiftly reversed with flumazenil, indicating significant potential for pediatric anesthesia (Buchwald, 2020; Kilpatrick et al., 2007). However, esketamine, a stereoisomer of ketamine, provides potent analgesic effects with mild sedative properties and is known for its protective airway reflexes and hemodynamic stability (Ihmsen et al., 2001; Turner, 2019). We hypothesized that combining remimazolam and esketamine may offer an effective strategy for endotracheal intubation in pediatric patients, potentially eliminating the need for traditional muscle relaxants.

Determining the optimal dosing of these agents is crucial to enhancing their therapeutic efficacy while minimizing potential adverse effects. However, studies on their use in pediatric anesthesia are limited, particularly regarding their application in intubation without muscle relaxants. This study investigated the effective doses of remimazolam and esketamine for endotracheal without muscular relaxation in pediatric patients aged 3–6 years.

## 2 Methods

### 2.1 Study design and participants

This prospective, non-controlled, non-randomized clinical trial employed Dixon’s up-and-down sequential allocation method. Approved by the Ethics Committee of the Affiliated Shunde Hospital of Jinan University (JDSY-LL-2022042, 02/04/2022), the trial was registered with the Chinese Clinical Trial Registry (www.chictr.org.cn, number: ChiCTR2200063847, 19/09/2022). Participants were recruited from the Hernia Surgery Department or the Otolaryngology Department of our hospital. All procedures strictly adhere to the relevant guidelines and regulations established by the Affiliated Shunde Hospital of Jinan University and comply with the Declaration of Helsinki and CIOMS guidelines, ensuring compliance with international ethical standards. Parents or guardians were provided with detailed information about the study’s risks and benefits, and written informed consent was obtained before enrollment.

### 2.2 Inclusion and exclusion criteria

Inclusion criteria: Participants were children aged 3 to 6, undergoing adenotonsillectomy or pediatric hydrocele surgery under general anesthesia with endotracheal intubation. They were classified as American Society of Anesthesiologists (ASA) physical status I or II, with a body mass index (BMI) of 13–22 kg/m^2^ and weighing 10–40 kg. There were no gender restrictions, and participants were normally developed.

Exclusion criteria: Allergy to remimazolam or esketamine, upper respiratory tract infection, severe heart and lung disease, airway hyperresponsiveness, and difficult airways.

### 2.3 Anesthesia procedure

The pediatric patients fasted for over 6 h and refrained from drinking clear liquids for at least 2 h before operation. Electrocardiogram, peripheral capillary oxygen saturation (SpO_2_), heart rate (HR), and mean arterial pressure (MAP) were monitored using a multifunction device upon entering the operating room. Patients received oxygen supplementation via a face mask at a flow rate of 3 L/min. The baseline of SpO_2_, HR, and MAP were measured 3 min after resting.

All pediatric patients were administered remifentanil (Jiangsu Enhua Pharmaceutical Co., China, diluted to 20 μg/mL with normal saline, lot number: TRF23I02) at a dose of 2.5 μg/kg using standard solution (pump, administered over 90 s) (Bai et al., 2024; Hirano et al., 2023). In the first phase, 0.2 mg/kg of remimazolam (Jiangsu Hengrui Medicine Co., China, diluted to 1 mg/mL with normal saline; lot number 220326 A K) was injected intravenously (no less than 10 s) in a fixed dose during anesthesia induction. Different dosages of esketamine (Jiangsu Hengrui Medicine Co., China, diluted to 2.5 mg/mL with normal saline; lot number 230908 B L) were administered using Dixon’s up-and-down sequential method. After intubation, analgosedation was maintained with a continuous infusion of remifentanil at 0.1–0.2 μg/kg/min and intermittent boluses of remimazolam (0.05–0.1 mg/kg) as needed to maintain adequate anesthesia depth. Esketamine was not administered post-intubation to avoid potential cumulative effects. In the first phase, the first patient received esketamine at 0.5 mg/kg, and subsequent patients were administered at a 0.2 mg/kg dose gradient. Once the eyelash reaction disappeared, indicating adequate anesthesia depth, an experienced anesthesiologist performed intubation using a visual laryngoscope, and the intubation response was evaluated. If the response to intubation was negative (effective), the dose gradient during induction was reduced for the subsequent case. Conversely, if the response was positive (ineffective), the dose gradient was increased. This process continued until the test was stopped after the occurrence of six positive and negative crosspoints (Pace and Stylianou, 2007). In the second phase, different dosages of remimazolam were administered intravenously, followed by a fixed dose of esketamine, which was the ED_95_ determined in the first phase. The first patient received 0.2 mg/kg of remimazolam, with subsequent doses administered in 0.1 mg/kg increments or decrements. The same assessment method used in the first phase was employed to evaluate the tracheal intubation and the response to intubation. Children were sequentially enrolled and assigned to receive either esketamine or remimazolam at varying doses based on the response of the previous patient, following Dixon’s up-and-down sequential allocation method.

### 2.4 Outcome assessments

Primary outcome measures: The endotracheal intubation response was assessed based on the Copenhagen scale (Viby-Mogensen et al., 1996). The Copenhagen scale is a validated tool for assessing intubation conditions, evaluating factors such as jaw relaxation, vocal cord visibility, and limb movement during intubation.

If the intubation response is negative, the drug dosage is effective, and the intubation conditions are satisfactory (Viby-Mogensen, et al., 1996). Specifically, this situation is reflected in the relaxation of the jaw during laryngoscopic intubation, allowing for smooth laryngoscopy, clear visibility of the vocal cords, and no coughing or limb movement responses. Additionally, the absence of responses to intubation stimuli means that the conditions for tracheal intubation have reached an excellent standard, with changes in MAP and HR before and after intubation being less than 20%. Conversely, if the intubation response is positive, it indicates that the drug dosage used is ineffective. If the mandible was stiff and the intubation condition was unsatisfactory, 0.2 mg/kg of mivacurium was added. If the MAP or HR increased by over 20% within 2 min after tracheal intubation, remimazolam was added to deepen anesthesia.

The secondary outcomes included HR and MAP measured at the following time points: Just before intubation (T1) and 1 min after intubation (T2). Hypotension, hypertension, bradycardia, tachycardia, SpO_2_ < 90%, bucking, mandibular stiffness, body movement, and glottal closure were recorded.

Adverse events were managed as follows: hypotension (MAP decreased by 20% or more from baseline) was treated with intravenous (IV) ephedrine 3–6 mg; hypertension (systolic pressure ≥140 mmHg or diastolic pressure ≥90 mmHg) was treated with IV remimazolam 0.1 mg/kg and sufentanil 5 μg; SpO_2_ < 90% was managed by applying positive pressure oxygen via a mask and increasing the depth of anesthesia; bradycardia (HR < 60 beats/min) was treated with IV atropine 0.2–0.3 mg; and tachycardia (HR exceeded 20% of baseline) was addressed with IV remimazolam 0.1 mg/kg or sufentanil 5 µg.

### 2.5 Statistical analysis

The sample size determination was performed using Dixon’s up-and-down method, requiring seven crosspoints (effective to ineffective) for statistical analysis (Pace and Stylianou, 2007). Statistical analyses were conducted using the Statistical Package for the Social Sciences (SPSS) software, version 26.0 (IBM Corp., Armonk, NY, United States). Data are presented as means ± standard deviation (SD) or as frequencies and percentages (n [%]). Comparisons between phases were conducted using the t-test, while categorical data were analyzed using the chi-square test or Fisher’s exact test. ED_50_ and ED_95_ of remimazolam and esketamine were determined through probit regression analysis, with a p-value of less than 0.05 considered indicative of statistical significance.

## 3 Results

### 3.1 Pediatric patients’ information

**FIGURE 1:**
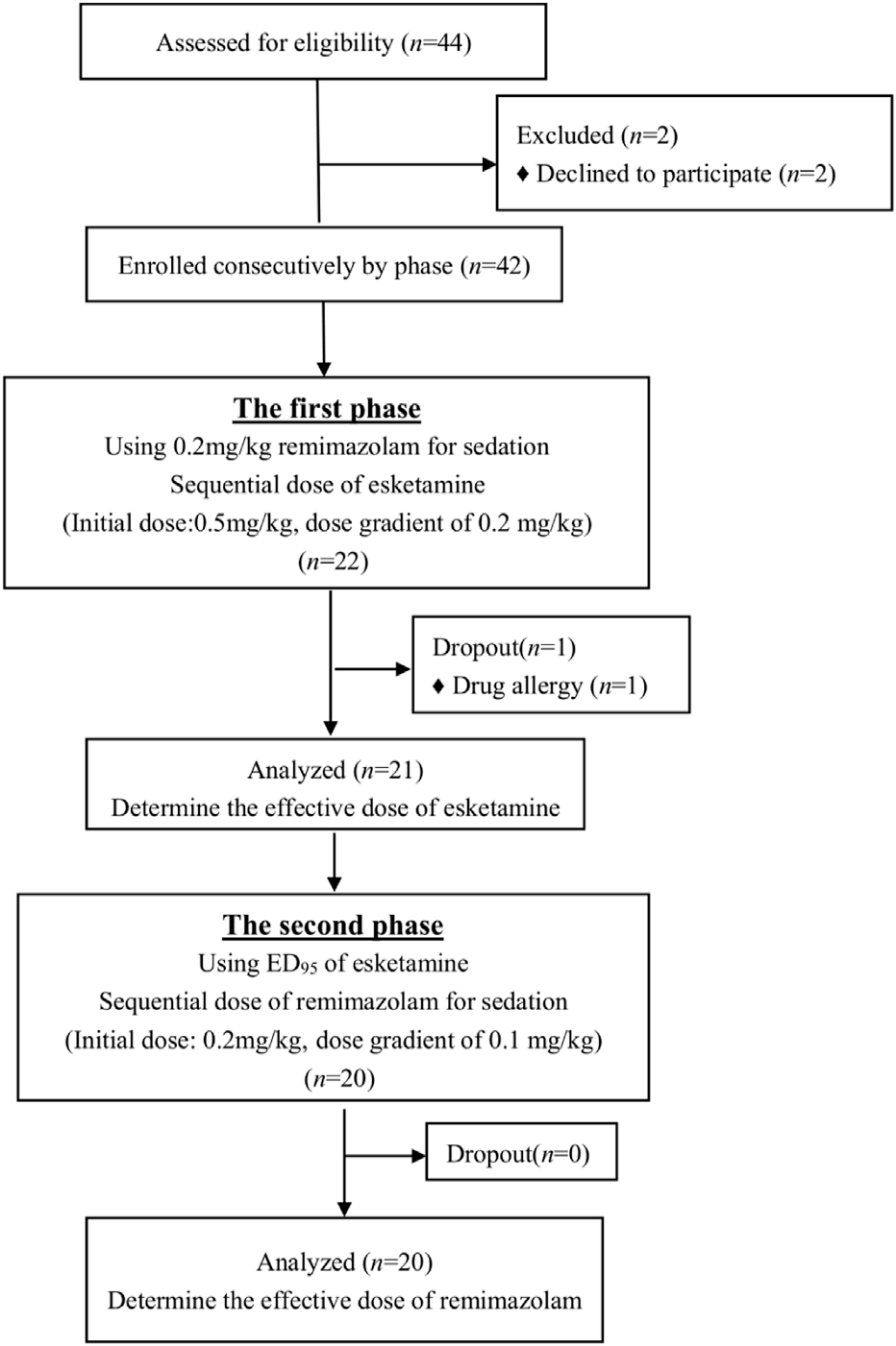
Flow diagram of the study.

**FIGURE 2:**
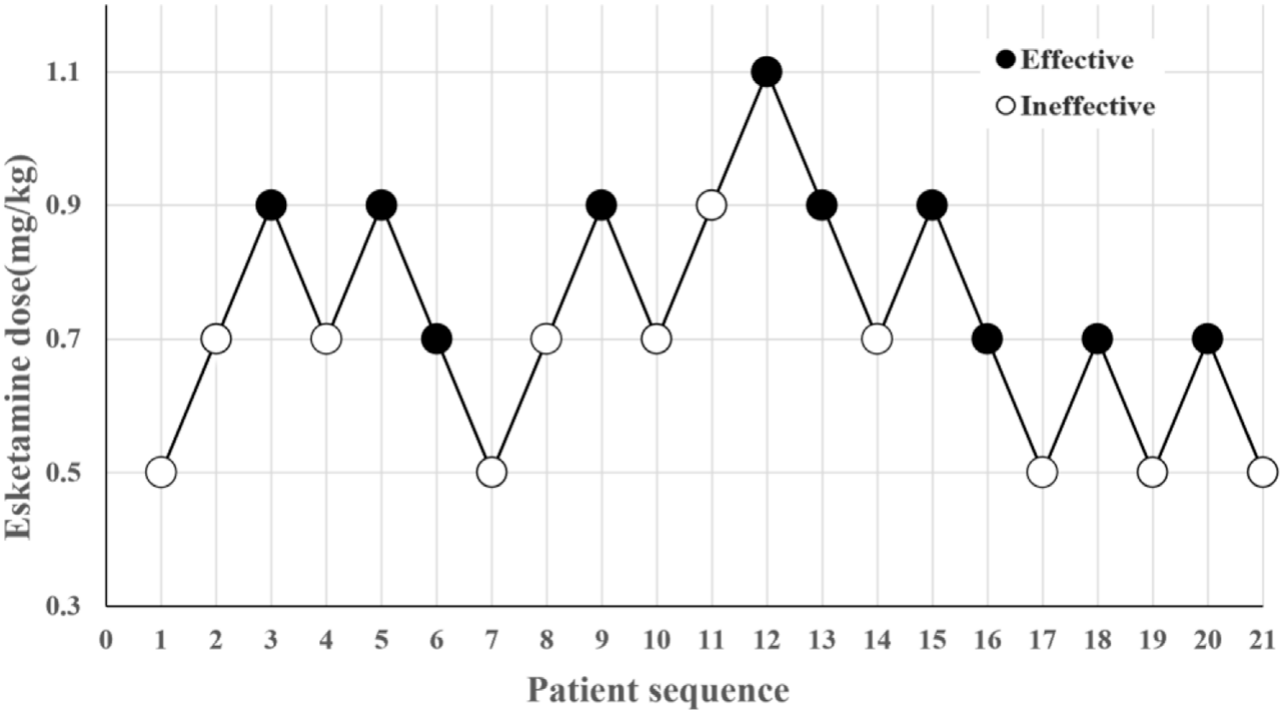
Sequential test of induced dose of esketamine.

**FIGURE 3:**
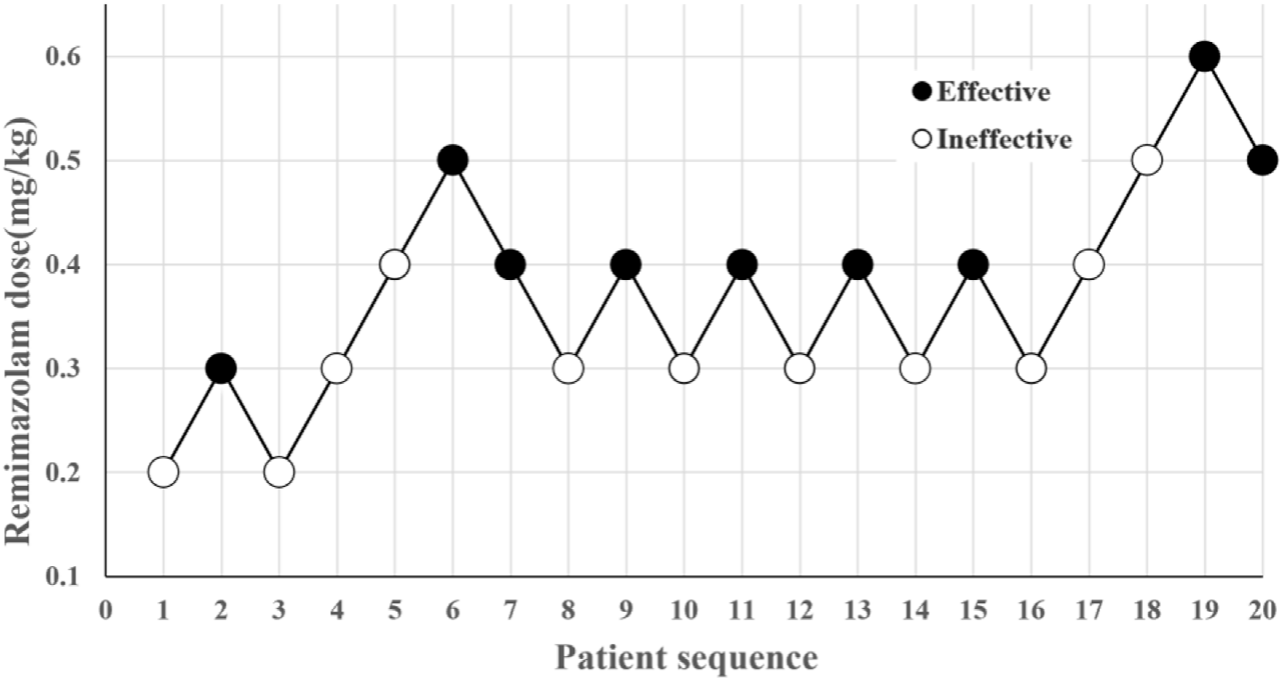
Sequential test of induced dose of remimazolam.

This study enrolled 44 pediatric patients across two phases and excluded 3 between January 2023 and February 2024. In the first phase, 21 pediatric patients were included for analysis, while 20 pediatric patients were included in the second phase, as displayed in the study flowchart (Figure 1). In the first phase, 10 cases were classified as “effective,” while 11 cases were identified as “ineffective” (Figure 2). In the second phase, 9 cases were classified as “effective” while 11 cases were identified as “ineffective” (Figure 3). There were no statistically significant differences in ASA, age, height, weight, and BMI among all patients (*p* > 0.05) as presented in Tables 1, 2.

**TABLE 1:** Patients’ characteristics in the first phase.

Characteristics	The first phase		*p*-value
	Effective group, n = 10	Ineffective group, n = 11	
ASA (I/II)	8/2	9/2	0.999
Age (years)	5.3 ± 1.3	5.2 ± 1.1	0.819
Height (cm)	113.9 ± 10.9	114.6 ± 12.3	0.892
Weight (kg)	18.4 ± 3.0	18.7 ± 3.8	0.828
BMI (kg/m^2^)	14.1 ± 1.1	14.2 ± 1.8	0.932

Data are expressed as means ± SD, or n.

**TABLE 2:** Patients’ characteristics in the second phase.

Characteristics	The second phase		*p*-value
	Effective group, n = 9	Ineffective group, n = 11	
ASA (I/II)	6/3	9/2	0.617
Age (years)	4.4 ± 1.3	4.3 ± 1.1	0.756
Height (cm)	108.7 ± 10.9	107.0 ± 7.8	0.695
Weight (kg)	19.8 ± 4.2	18.1 ± 4.1	0.388
BMI (kg/m^2^)	17.4 ± 3.1	15.7 ± 1.9	0.169

Data are expressed as SD, or n.

### 3.2 Effective dose of remimazolam and esketamine in both phases

**FIGURE 4:**
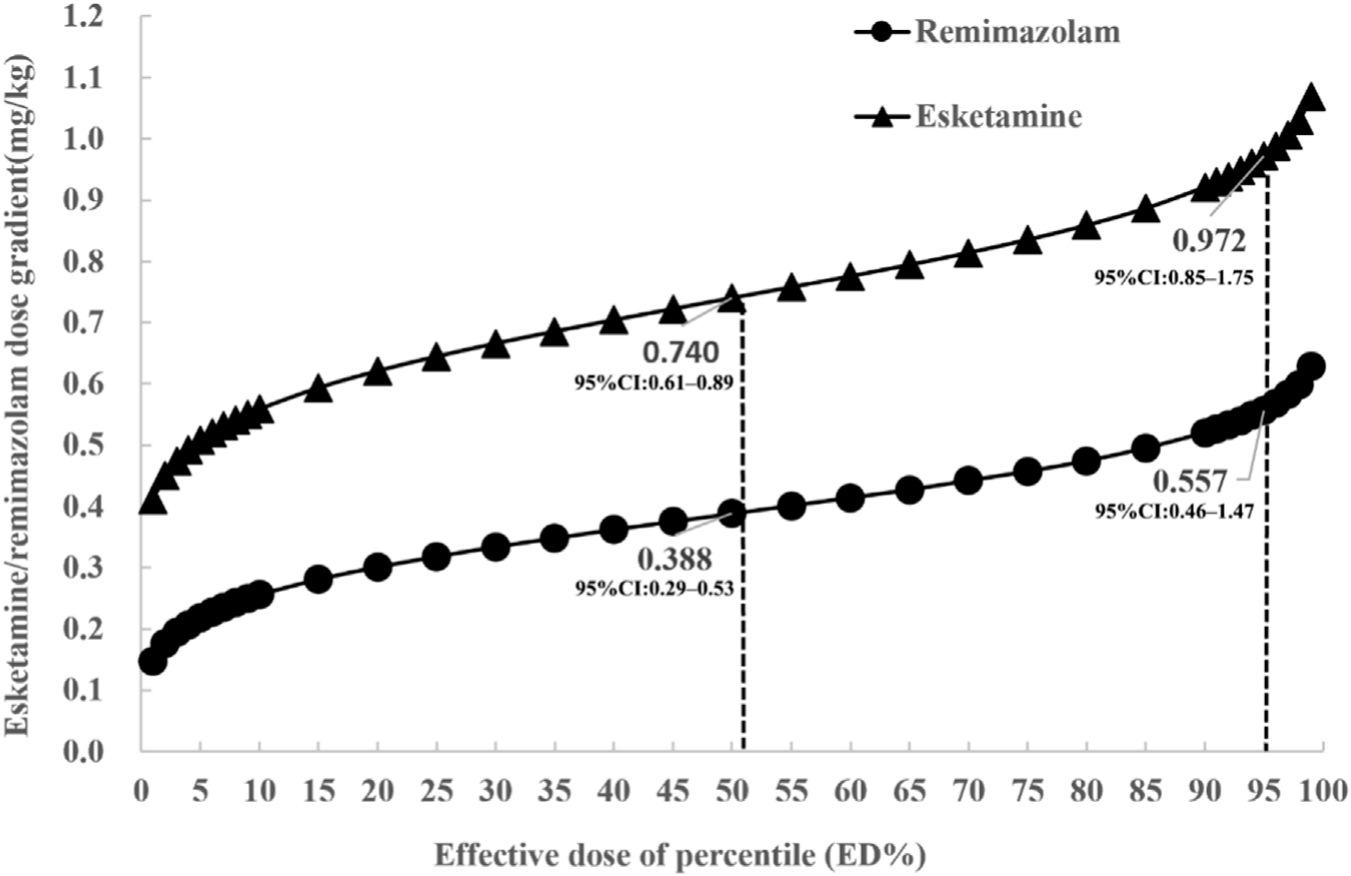
Dose⁃effect analysis of the induction doses of esketamine and remimazolam on inhibition of tracheal intubation response in both phases.

Probit regression analysis, based on a fixed dose of 2.5 μg/kg remifentanil, revealed that the ED_50_ of esketamine was 0.74 mg/kg (95% confidence interval [CI]: 0.61–0.89 mg/kg) and the ED_95_ was 0.97 mg/kg (95% CI: 0.85–1.75 mg/kg) in the first phase. Based on the ED_95_ of esketamine in the first phase, the ED_50_ of remimazolam was 0.39 mg/kg (95% CI: 0.29–0.53 mg/kg), and the ED_95_ was 0.56 mg/kg (95% CI: 0.46–1.47 mg/kg) (Figure 4).

### 3.3 Hemodynamic changes in both phases

The HR increased significantly at T2 in both phases (*p* < 0.05), this may be related to the sympathetic excitatory effect of esketamine and the stimulation of intubation. In the second phase, the MAP at T2 significantly increased compared to T1 (*p* = 0.006), whereas no significant differences in MAP were observed between T1 and T2 in the first phase (*p* = 0.269), as illustrated in Table 3. This difference may be attributed to the higher dose of esketamine used in the second phase, which could have led to greater sympathetic stimulation and subsequent elevation in blood pressure.

**TABLE 3:** Comparison of HR and MAP between the both phases at different time points.

Index	Phase	Time point		*p*-value
		T1	T2	
HR (beats/min)	First	109.7 ± 19.8	123.9 ± 17.1	0.017
	Second	102.6 ± 11.1	123.9 ± 18.5	0.000
MAP (mmHg)	First	78.7 ± 9.7	82.8 ± 13.4	0.269
	Second	74.6 ± 11.9	86.3 ± 13.3	0.006

Data are expressed as mean ± SD.

T1, just before intubation; T2, At 1 min after intubation.

No statistically significant differences in HR and MAP between the phases at T1 and T2 time point (*p* > 0.05).

### 3.4 Adverse events in both phases

No significant difference was observed in the incidence of adverse events during anesthesia induction (*p* > 0.05) (Table 4). However, the incidence of tachycardia (85.7% in Phase 1 and 85% in Phase 2) highlights the need for careful monitoring of HR during anesthesia induction.

**TABLE 4:** Adverse events during anesthesia induction.

Adverse events	The first phase, n = 21	The second phase, n = 20	*P* -value
Hypotension	0 (0)	0 (0)	—
Hypertension	3 (14.3)	2 (10)	0.684
Bradycardia	0 (0)	0 (0)	—
Tachycardia	18 (85.7)	17 (85)	0.950
SpO_2_ < 90%	0 (0)	0 (0)	—
Bucking	5 (23.8)	9 (45)	0.197
Mandibular stiffness	0 (0)	0 (0)	—
Body movement	5 (23.8)	5 (25)	>0.999
Glottal closure	2 (9.5)	4 (20)	0.410

Data are expressed as n [%].

## 4 Discussion

Based on a fixed dose of 2.5 μg/kg remifentanil, ED_50_/ED_95_ of esketamine was 0.74 mg/kg (95% CI:0.61–0.89) and 0.97 mg/kg (95% CI: 0.85–1.75), respectively. Similarly, ED_50_/ED_95_ of remimazolam was 0.39 mg/kg (95% CI: 0.29–0.53) and 0.56 mg/kg (95% CI: 0.46–1.47), respectively. These findings can provide initial dosing references for children aged 3–6 undergoing tracheal intubation without muscle relaxation.

The results indicated that during the second phase, MAP at T2 was significantly higher than at T1 (*p* < 0.05), whereas no significant differences in MAP were observed between T1 and T2 in the first phase (*p* > 0.05). The possible reason is that the second phase involved administering the ED_95_ of esketamine, which was marginally higher than the dose used in the first phase. Moreover, esketamine has the property of stabilizing hemodynamic parameters (Del Sant et al., 2020; Zhao et al., 2024), especially in pediatric cardiovascular anesthesia (Shimizu et al., 2024). In this study, the effect of esketamine on HR increased with dosage; however, its impact on blood pressure was not evident, possibly due to its effects on the sympathetic nervous system and catecholamine levels (Mihaljević et al., 2020). Although the incidence of tachycardia exceeded 80% in both phases and remained manageable without special treatment, these findings highlight the importance of continuous hemodynamic monitoring when using this drug combination in pediatric patients. Remifentanil primarily suppresses HR, while esketamine increases HR and bronchial relaxation (Zanos et al., 2018). Using a low dose of remifentanil reduces pharyngeal reflexes and sympathetic nerve stimulation during intubation (Kang et al., 2022), which helps avoid side effects from increased dosages of esketamine and remimazolam. This drug regimen, comprising fast-acting, short-duration medications, promotes quick recovery in children, prevents the onset of hyperalgesia (increased sensitivity to pain), and enhances patient comfort (Colvin et al., 2019). However, it is crucial to recognize that the safety results in this study are secondary and should be viewed cautiously because of the relatively small sample size. While no patient required treatment for bradycardia or hypotension during the trial, further studies with larger cohorts are needed to confirm the safety profile of this drug combination.

This study found that six children (two in the first phase and four in the second phase) experienced glottal closure during intubation. The symptoms resolved after deepening the anesthesia and administering muscle relaxants, indicating that using anesthesia without muscle relaxants in children can be risky. A research revealed that hypoxia and laryngospasm constitute approximately 30% of respiratory events, with intubation difficulties accounting for 13% and bronchospasm for 7% during pediatric anesthesia (Von Ungern-Sternberg and Habre, 2007). Consequently, implementing appropriate preventive and therapeutic measures in pediatric anesthesia is imperative.

Although the ED_95_ of esketamine in this study is larger than that reported in previous studies (Su et al., 2023; Wang et al., 2022; Zheng et al., 2023; Zheng et al., 2022; Zhong et al., 2023), it still falls within the recommended safe range of 0.5–1 mg/kg for induction and maintenance anesthesia (Kohtala, 2021; Trimmel et al., 2018). Possible reasons include: (1) Compared to propofol, remimazolam has a milder cardiovascular depressant effect, and its overall efficacy is lower (Fang et al., 2023); (2) the dosage requirements of esketamine can differ among children in various age groups (Leroux et al., 2021); (3) the effective dose of esketamine was determined by administering a relatively small dose of remimazolam at 0.2 mg/kg. Consequently, despite being slightly larger than expected, the determined ED_95_ for esketamine at 0 0.97 mg/kg (95% CI: 0.85–1.75 mg/kg) remains credible and suitable for clinical application.

The standard sample size for the modified sequential method is 20–40 cases (Wei et al., 2015). This test included over 20 cases in each stage, fully complying with the testing methodology’s requirements. The primary objective of this study was to assess the changes in intubation conditions and hemodynamics both before and after intubation, regardless of the surgical. The study results revealed that no significant circulatory inhibition was observed with increased dosages of esketamine. Moreover, a higher dose of remimazolam increased effectiveness in inhibiting reactions to endotracheal intubation. However, lower doses of remimazolam resulted in elevated reactions, such as body movement and coughing, and substantial fluctuations in MAP and HR before and after intubation.

This study has several limitations. First, our study focused on children aged 3–6 years, and the dosing requirements may differ for younger or older pediatric patients. Younger children, for example, may require lower doses due to differences in drug metabolism, while older children may need higher doses to achieve the same effect. Future studies should explore the dosing requirements across a broader age range to provide more clinical practice recommendations. Second, lack of bispectral index monitoring for anesthesia depth is a notable limitation, as it could have provided more precise data on the depth of sedation during intubation. Finally, while our findings provide valuable insights, the single-center design may limit the generalizability of our findings to other settings with different patient populations or clinical practices. Future multicenter studies with larger cohorts are needed to validate these findings and make them applicable to a larger group of children.

In conclusion, the ED_50_ and ED_95_ values of remimazolam and esketamine determined in this study provide initial dosing references for clinical practice. Administering a fixed dose of 2.5 μg/kg remifentanil combined with these agents can be safely and effectively used for endotracheal intubation without muscle relaxants in pediatric patients aged 3–6 years. However, further validation through larger, multicenter studies is recommended.

## Data Availability

The raw data supporting the conclusions of this article will be made available by the authors, without undue reservation.
